# Development and internal temporal validation of an explainable XGBoost model for predicting clinical pregnancy after frozen embryo transfer

**DOI:** 10.3389/fendo.2026.1942415

**Published:** 2026-09-28

**Authors:** Xudong Hu, Yanchun Zhao, Yunping Ni, Aiai Wang, Juan Liu, Yanyu Zhao, Xinghong Lu

**Affiliations:** 1 The Second Clinical College of Guangzhou University of Chinese Medicine, Guangzhou, Guangdong, China; 2 Ludwig-Maximilians-Universität München, Munich, Germany

**Keywords:** clinical pregnancy, frozen embryo transfer, predictive model, Shapley additive explanations, XGBoost

## Abstract

**Key message:**

An explainable machine learning model was developed using comprehensive clinical variables to predict clinical pregnancy outcomes following frozen embryo transfer. Compared with conventional statistical models, the extreme gradient boosting–based model showed numerically higher discrimination and provided interpretable insights through SHapley Additive exPlanations (SHAP) analysis, supporting individualized decision-making in reproductive medicine.

**Research question:**

Can an interpretable XGBoost-based model leveraging multi-feature clinical data better predict pregnancy outcomes after frozen embryo transfer (FET) compared to conventional logistic regression?

**Design:**

This retrospective single-center study included 1,319 patients who underwent FET. Participants were temporally split into a training cohort (n=1,013) and a temporal validation cohort (n=306) according to the date of embryo transfer. Pre-transfer demographic, hormonal, treatment, and ultrasonographic variables were collected. An XGBoost model was developed and compared with logistic regression by assessing discrimination (area under the ROC curve [AUC] and area under the precision–recall curve [AUPRC]), calibration (calibration curves, Brier score, intercept, and slope), and clinical utility using decision curve analysis (DCA). Model interpretability was evaluated using SHapley Additive exPlanations (SHAP).

**Results:**

The XGBoost model achieved an AUC of 0.854 (95% CI 0.832–0.877) in training and 0.722 (95% CI 0.666–0.779) in temporal validation, with corresponding AUPRC values of 0.840 and 0.707. Calibration in the temporal validation cohort was acceptable (Brier score 0.212; calibration intercept 0.056; slope 0.965). DCA indicated a positive net benefit across clinically relevant thresholds, with high sensitivity at a representative probability threshold of 0.30. SHAP visualizations provided feature-level and individual-level explanations.

**Conclusion:**

Using single-center data, we developed and internally, temporally validated an explainable XGBoost model for predicting clinical pregnancy after FET. The model delivers accurate, interpretable predictions that may support personalized decision-making and facilitate the clinical integration of artificial intelligence into assisted reproductive medicine.

## 1 Introduction

Infertility represents a growing global public health concern. According to recent estimates from the World Health Organization (WHO), approximately one in six individuals of reproductive age worldwide experiences infertility at some point in their lives (1). Driven by lifestyle and environmental factors, infertility rates have continued to rise globally, particularly in some regions (2). The advancement of assisted reproductive technologies (ART), particularly *in vitro* fertilization (IVF) and intracytoplasmic sperm injection (ICSI), has markedly improved therapeutic options for infertility management. Frozen embryo transfer (FET) has become increasingly prevalent in clinical practice, as it reduces the risk of ovarian hyperstimulation syndrome, enhances endometrial receptivity, and provides flexibility in transfer scheduling (3). According to the latest data from the European Society of Human Reproduction and Embryology (ESHRE), FET cycles comprise 36.3% of all ART treatments and continue to increase (4), yet the clinical pregnancy rate remains only about 33-37% (5), rendering outcome prediction challenging owing to the multifactorial nature of reproductive success.

The variables influencing pregnancy outcomes following FET remain insufficiently understood, and the predictive performance of existing models remains suboptimal (6–8). Although conventional multivariable regression models have been widely applied in reproductive medicine, they are inadequate for capturing complex nonlinear relationships and interactions among variables, thereby constraining further improvements in predictive accuracy. With the rapid advancement of artificial intelligence (AI), machine learning (ML) techniques have been increasingly applied to clinical prediction research. Among these, eXtreme Gradient Boosting (XGBoost) has demonstrated superior accuracy and generalizability across various medical prediction scenarios (9). However, XGBoost is considered a typical “black-box” model, with an internal decision-making process that is difficult to interpret intuitively (10, 11), which limits its clinical applicability. Recently, the emergence of explainable artificial intelligence (XAI) techniques (12) has provided new solutions to this challenge, among which the Shapley Additive exPlanations (SHAP) method (13) quantifies the marginal contribution of each factor to the prediction, clarifying the model’s decision logic and enabling interpretability while maintaining high predictive performance.

Therefore, using single-center data, this study aimed to develop and temporally validate an explainable XGBoost-based prediction model with SHAP to evaluate clinical pregnancy outcomes following FET. The performance was compared with that of a conventional logistic regression model to provide an evidence-based reference for individualized pregnancy prediction and clinical decision-making in assisted reproduction.

## 2 Materials and methods

### 2.1 Study population

This retrospective study included patients who underwent FET in the Department of Reproductive Medicine at a large tertiary reproductive center, Guangdong Provincial Hospital of Chinese Medicine, between March 1, 2023 and March 1, 2025. The study protocol was approved by the Ethics Committee of the Guangdong Provincial Hospital of Chinese Medicine (Approval number: ZE2025-398-01) and was conducted in accordance with the principles of the Declaration of Helsinki. Given its retrospective design, all patient data were anonymized to ensure confidentiality and informed consent was waived by the ethics committee. The study was reported in accordance with the TRIPOD+AI statement for prediction model studies (14).

Inclusion criteria: patients who underwent complete FET at our center; had available clinical pregnancy follow-up data; and for those with multiple FET cycles during the study period, only the first eligible cycle was included.

Exclusion criteria: patients with uterine malformations (such as bicornuate or rudimentary uterus) causing significant distortion of the uterine cavity; had substantial missing clinical information (missing data > 20%).

As this was a retrospective single-center study, the sample size was determined by the number of eligible patients during the study period. The final cohort provided a sufficient number of outcome events relative to the predictors included, supporting the adequacy of the sample size for model development.

### 2.2 Clinical data collection

Data were independently extracted and cross-validated by two researchers using a double-entry verification approach. Any discrepancies were resolved by a third investigator, who verified the final records. All data were obtained from the electronic medical record system and the assisted reproduction management platform of the Department of Reproductive Medicine, Guangdong Provincial Hospital of Chinese Medicine.

This study collected four categories of clinical variables: demographic information (e.g., age and body mass index [BMI]); infertility-related history (e.g., infertility type, previous reproductive history, and gynecological disorders); assisted reproduction–related parameters (e.g., fertilization method, cycle type, and embryo transfer characteristics); laboratory and imaging indicators (e.g., AMH level, antral follicle count [AFC], and endometrial parameters).

### 2.3 Definition of data and variable transformation

#### 2.3.1 Endometrial preparation protocol

Based on menstrual and ovulatory status, five types of endometrial preparation cycles were used: Natural cycle (NC), Hormone replacement therapy cycle (HRT), Ovulation induction (OI), GnRH-a-HRT, GnRH-a-OI. Detailed protocols are provided in the **Supplementary Material (Part 1)**.

#### 2.3.2 Endometrial thickness change

Recent studies have suggested that a single measurement of endometrial thickness (ET) may not fully reflect its dynamic changes in endometrial receptivity (15, 16). Therefore, a new variable “endometrial change” was established to quantify the stability and trend of the endometrium from progesterone initiation to embryo transfer. It was defined as the difference between embryo transfer–day ET and Pre-progesterone ET, and was categorized into three groups according to the difference: (1) Decrease (≤ −1 mm); (2) Stable (−1 mm to +1 mm); and (3) Increase (≥ +1 mm).

#### 2.3.3 Categorization of continuous variables

Because conventional regression models may not fully capture nonlinear relationships between continuous predictors and outcomes, several continuous variables were categorized based on clinical relevance to improve discriminative performance, including BMI, ET, and AFC. Detailed grouping criteria are provided in Data dictionary for candidate predictors (**Supplementary Material Part, 2**).

### 2.4 Study outcomes and evaluation methods

Clinical pregnancy, the primary outcome of this study, was defined as the presence of a gestational sac (intrauterine or ectopic) (17), detected by transvaginal ultrasound after embryo transfer. All patients underwent transvaginal ultrasonography 5–7 weeks after transfer, and the images were independently reviewed by two reproductive specialists to confirm clinical pregnancy status. Ectopic pregnancies were included in the composite clinical pregnancy outcome and were reported separately in the Results section.

### 2.5 Statistical analysis

Statistical analyses were performed in R (version 4.4.2) (18), using packages including XGBoost, missForest, ggplot2, pROC, and shapviz. Missing data were assessed at the variable level. Variables with a missing rate greater than 3% included anti-Müllerian hormone (AMH; 58 cases, 4.4%), endometrial thickness on the progesterone day (81 cases, 6.1%), and endometrial thickness on the embryo transfer day (78 cases, 5.9%). Missing values were imputed using a random forest–based approach, fitted on the training set only and applied to the validation set through a locked preprocessing pipeline. Continuous variables were tested for normality. Variables with a normal distribution were expressed as mean ± standard deviation (SD) and compared between groups using the independent-samples t test, whereas non-normally distributed variables were expressed as median (interquartile range) [M (P25, P75)] and compared using the Mann–Whitney U test. Categorical variables were presented as frequencies and percentages, and group differences were evaluated using the chi-square test or Fisher’s exact test (when the expected frequency was <5 or the sample size was small). A two-sided *P* value <0.05 was considered statistically significant.

#### 2.5.1 Variable selection and model development

Patients who completed embryo transfer from March 1, 2023 to September 30, 2024 were included in the training cohort, with all variables considered as candidate predictors. Feature selection was performed using the LASSO regression with 10-fold cross-validation to minimize overfitting (19). The events-per-parameter ratio was calculated based on the number of clinical pregnancy events in the training cohort and the number of estimated predictor parameters excluding the intercept. The generalized variance inflation factors (GVIFs) were calculated to assess multicollinearity among variables. The selected variables were used to develop two predictive models: a conventional logistic regression model (visualized as a nomogram) and an XGBoost model. Detailed model specifications and parameter settings are described in **Supplementary Material (Part 3)**. To improve the interpretability of the XGBoost model, feature importance was illustrated using bar plots of mean absolute SHAP values, whereas SHAP beeswarm plots were utilized to demonstrate the direction and magnitude of effects, nonlinear associations, and interindividual heterogeneity across variables. XGBoost complexity was characterized by its tree structure and key hyperparameters. The final model used a learning rate of 0.1, a maximum tree depth of 3, and 100 boosting trees.

#### 2.5.2 Evaluation of model performance and clinical utility

The discriminative performance of both the nomogram and the XGBoost model was primarily assessed by calculating the areas under the receiver operating characteristic (ROC) curves (AUCs), together with the area under the precision–recall curve (AUPRC). Internal validation was performed using the bootstrap resampling method with 1000 iterations to estimate 95% confidence intervals. Model calibration was assessed using calibration curves, the Brier score, and calibration intercept and slope. Decision curve analysis (DCA) was conducted to evaluate the clinical utility of the models, and performance at a clinically representative probability threshold (0.30) was additionally examined to aid interpretation. To further evaluate the generalizability of the models in real-world clinical settings, patients who underwent embryo transfer between October 1, 2024 and March 1, 2025 were included as a temporal validation cohort, and the AUC, AUPRC, calibration, and DCA were recalculated. The temporal validation cohort was used exclusively for performance evaluation and was not involved in any stage of model development, feature selection, or hyperparameter tuning.

## 3 Results

According to the inclusion and exclusion criteria, a total of 1,319 patients who underwent their first FET at our center were included. (detailed selection process in **Supplementary Material, Part 4**). Among them, 1,013 were assigned to the training cohort and 306 to the temporal validation cohort. Clinical pregnancy was achieved in 642 patients, including 11 cases of ectopic pregnancy. Significant differences were observed between the two cohorts in infertility type, history of cesarean section, number of transferred embryos, number of good-quality embryos, diminished ovarian reserve (DOR), and adverse reproductive history, while other baseline characteristics were generally comparable (**Table 1**). The outcome distribution was approximately balanced (about 50% clinical pregnancy), therefore no class imbalance correction was applied.

**Table 1:** Baseline characteristics of participants in the training and validation csohorts.

Variables	Total (n=1319)	Train (n=1013)	validation (n=306)	*P*
Age (years),Median (Q1,Q3)	34 (31,37)	33 (31,37)	35 (31,37)	0.154
BMI,Median (Q1,Q3)	21.83 (19.93,24.03)	21.78 (19.93,24)	22 (19.99,24.3)	0.319
AMH,Median (Q1,Q3)	3 (1.49,4.69)	3.02 (1.53,4.72)	2.79 (1.29,4.51)	0.109
AFC,Median (Q1,Q3)	14 (8,22)	15 (8,22)	14 (7.25,21.75)	0.103
duration of infertility (years),Median (Q1,Q3)	2 (1,4)	2.5 (1,4)	2 (1.5,4)	0.765
Infertility type,n (%)				0.033*
Secondary infertility	630 (48)	467 (46)	163 (53)	
Primary infertility	689 (52)	546 (54)	143 (47)	
History of caesarean section,n (%)				0.010*
0	1131 (86)	883 (87)	248 (81)	
1	188 (14)	130 (13)	58 (19)	
Fertilization method,n (%)				0.411
ICSI	345 (26)	271 (27)	74 (24)	
IVF	974 (74)	742 (73)	232 (76)	
Induced abortions,n (%)				0.098
0	956 (72)	746 (74)	210 (69)	
1	259 (20)	193 (19)	66 (22)	
2	66 (5)	46 (5)	20 (7)	
3	26 (2)	21 (2)	5 (2)	
≥4	12 (1%)​	7 (1)​	5 (2)​	
Cycle type,n (%)				0.073
GnRH-a-HRT	667 (51)	499 (49)	168 (55)	
GnRH-a-OI	40 (3)	28 (3)	12 (4)	
OI	272 (21)	208 (21)	64 (21)	
HRT	156 (12)	132 (13)	24 (8)	
Naturalcycle (NC)	184 (14)	146 (14)	38 (12)	
Retrieval-transfer interval (months),Median (Q1,Q3)	5 (2,14)	5 (2,14)	5 (2.25,13)	0.709
Transferred embryos,n (%)				0.019*
1	959 (73)	720 (71)	239 (78)	
2	360 (27)	293 (29)	67 (22)	
Good-quality embryos,n (%)				0.006*
0	280 (21)	234 (23)	46 (15)	
1	948 (72)	707 (70)	241 (79)	
2	91 (7)	72 (7)	19 (6)	
Pre-progesterone ET (mm),Median (Q1,Q3)	9.4 (8.3,11)	9.5 (8.4,11)	9.3 (8.1,11)	0.381
Embryo transfer–day ET (mm),Median (Q1,Q3)	10 (8.6,11.8)	10 (8.7,12)	9.9 (8.2,11.28)	0.065
Endometrial change,n (%)				0.465
Decrease	204 (15)	160 (16)	44 (14)	
Stable	679 (51)	512 (51)	167 (55)	
Increase	436 (33)	341 (34)	95 (31)	
Polycystic ovary syndrome (PCOS),n (%)				0.120
0	1116 (85)	848 (84)	268 (88)	
1	203 (15)	165 (16)	38 (12)	
Endometriosis,n (%)				0.303
0	1109 (84)	858 (85)	251 (82)	
1	210 (16)	155 (15)	55 (18)	
Sequelae of pelvic inflammatory disease (PID),n (%)				0.228
0	821 (62)	640 (63)	181 (59)	
1	498 (38)	373 (37)	125 (41)	
Adverse reproductive history,n (%)				0.003*
0	1109 (84)	869 (86)	240 (78)	
1	210 (16)	144 (14)	66 (22)	
Diminished ovarian reserve (DOR),n (%)				0.016*
0	1108 (84)	865 (85)	243 (79)	
1	211 (16)	148 (15)	63 (21)	
Tubal abnormality,n (%)				0.267
0	1119 (85)	866 (85)	253 (83)	
1	200 (15)	147 (15)	53 (17)	
Pregnancy status,n (%)				0.738
0	677 (51)	523 (52)	154 (50)	
1	642 (49)	490 (48)	152 (50)	

*p.value≤0.05.

### 3.1 Variable selection

All candidate predictors were incorporated into the LASSO regression model for variable selection. The results showed that the model achieved optimal performance when λ = 0.015, identifying the set of predictors with the minimum nonzero coefficients (**Figure 1**). The final variables included in the model were age, induced abortions, cycle type, good-quality embryos, transferred embryos, endometrial change, BMI, AFC, embryo transfer-day ET, and retrieval-transfer interval. In the training cohort, 490 clinical pregnancy events were observed. The final logistic regression model contained 27 estimated predictor parameters excluding the intercept, corresponding to an events-per-parameter ratio of approximately 18.1, suggesting an adequate number of outcome events relative to the number of estimated parameters. The multicollinearity analysis showed that the GVIF^1/(2*df) values for all variables ranged from 1.023 to 1.291, which were far below the commonly accepted thresholds for multicollinearity (GVIF ^1/(2*df)> 5 or > 10), indicating no substantial multicollinearity among the included predictors.

**Figure 1:**
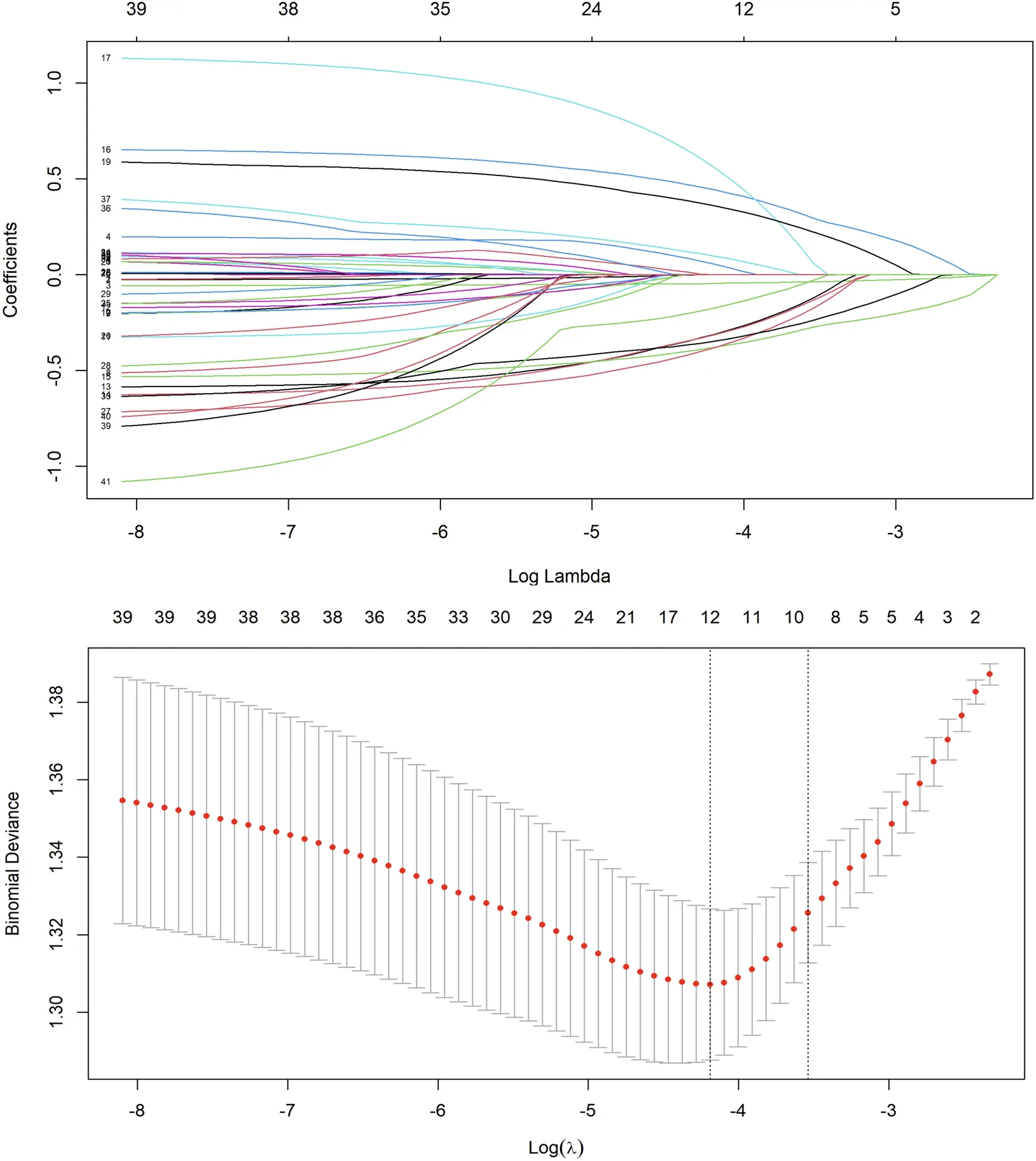
LASSO regression for feature selection with cross-validation.

### 3.2 Development and validation of the conventional nomogram model

Multivariate logistic regression was performed using the final variables (**Table 2**), to develop a clinical pregnancy prediction model presented as a nomogram (**Figure 2**). For illustration, a 33-year-old woman had a BMI of 18.1 kg/m², underwent an OI cycle, had one induced abortion, two good-quality embryos, two transferred embryos, stable endometrial change, an AFC of 19, a pre-progesterone ET of 11 mm, and a retrieval–transfer interval of 42 months. Summing the specific points on the nomogram yielded a total score of approximately 252, corresponding to a predicted clinical pregnancy probability of approximately 51%. In the training and temporal validation cohorts, the model achieved AUCs of 0.705 (95% CI, 0.675–0.738) and 0.690 (95% CI, 0.630–0.748), indicating moderate discriminative ability (**Figures 3A, B**). The corresponding AUPRC values were 0.687 and 0.650, respectively.

**Table 2:** Multivariate logistic regression analysis of clinical pregnancy risk.

Variables	β	S.E.	Z	OR	p.value
Age(years)	-0.057	0.017	-3.398	0.94 (0.91-0.98)	0.001*
Induced abortions					
0	0			1(Reference)	
1	0.270	0.181	1.494	1.31 (0.92-1.87)	0.135
2	0.099	0.332	0.297	1.10 (0.58-2.12)	0.766
3	0.181	0.488	0.37	1.20 (0.46-3.12)	0.711
4	-0.076	1.201	-0.064	0.93 (0.09-9.76)	0.949
5	-0.320	1.315	-0.243	0.73 (0.06-9.57)	0.808
Cycle type					
GnRH-a-HRT	0			1(Reference)	
GnRH-a-OI	-0.291	0.426	-0.682	0.75 (0.32-1.72)	0.495
HRT	-0.591	0.219	-2.7	0.55 (0.36-0.85)	0.007*
NC	-0.601	0.207	-2.899	0.55 (0.37-0.82)	0.004*
OI	-0.192	0.180	-1.068	0.83 (0.58-1.17)	0.286
Good-quality embryos					
0	0			1(Reference)	
1	0.666	0.190	3.502	1.95 (1.34-2.83)	<0.001*
2	1.163	0.310	3.749	3.20 (1.74-5.88)	<0.001*
Endometrial change					
Decrease	0			1(Reference)	
Increase	0.567	0.217	2.614	1.76 (1.15-2.70)	0.009*
Stable	0.019	0.201	0.096	1.02 (0.69-1.51)	0.924
Transferred embryos					
1	0			1(Reference)	
2	-0.524	0.197	-2.658	0.59 (0.40-0.87)	0.008*
BMI					
<18.5	0			1(Reference)	
>30	0.159	0.526	0.302	1.17 (0.42-3.29)	0.763
18.5–25	0.583	0.230	2.535	1.79 (1.14-2.81)	0.011*
25–30	0.651	0.283	2.297	1.92 (1.10-3.34)	0.022*
AFC					
<10	0			1(Reference)	
>30	0.411	0.239	1.718	1.51 (0.94-2.41)	0.086
10–20	0.431	0.173	2.484	1.54 (1.10-2.16)	0.013*
20–30	0.580	0.219	2.646	1.79 (1.16-2.75)	0.008*
Pre-progesterone ET (mm)					
<7	0			1(Reference)	
>12	0.417	0.366	1.138	1.52 (0.74-3.11)	0.255
10-12	0.466	0.315	1.483	1.59 (0.86-2.95)	0.138
7–10	0.189	0.290	0.654	1.21 (0.69-2.13)	0.513
Retrieval-transfer interval (months)					
<10	0			1(Reference)	
>60	0.727	0.352	2.063	2.07 (1.04-4.12)	0.039*
10–30	-0.300	0.202	-1.481	0.74 (0.50-1.10)	0.139
30–60	-0.020	0.224	-0.089	0.98 (0.63-1.52)	0.929

*p.value≤0.05.

**Figure 2:**
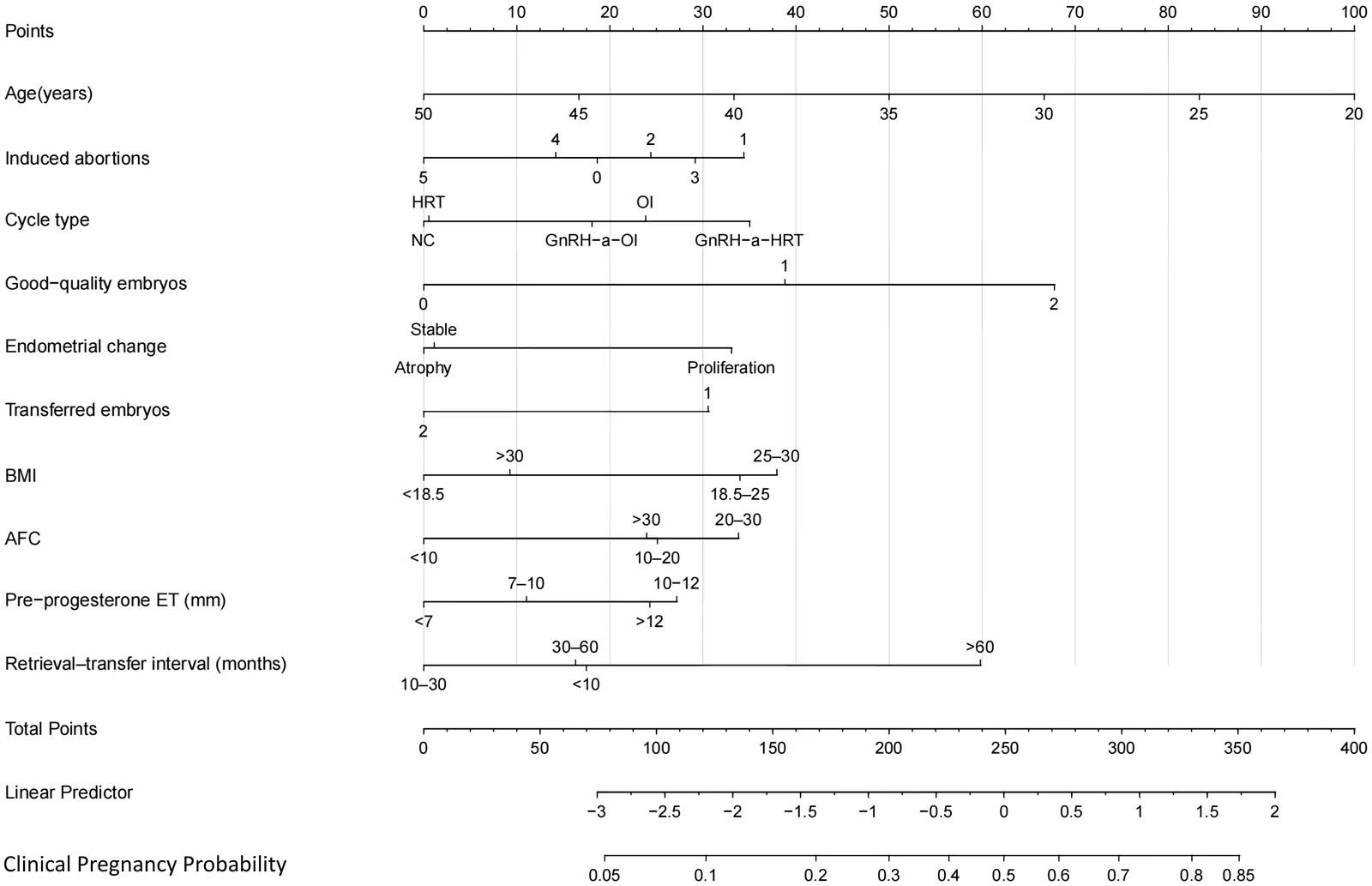
Nomogram for predicting clinical pregnancy after FET.

**Figure 3:**
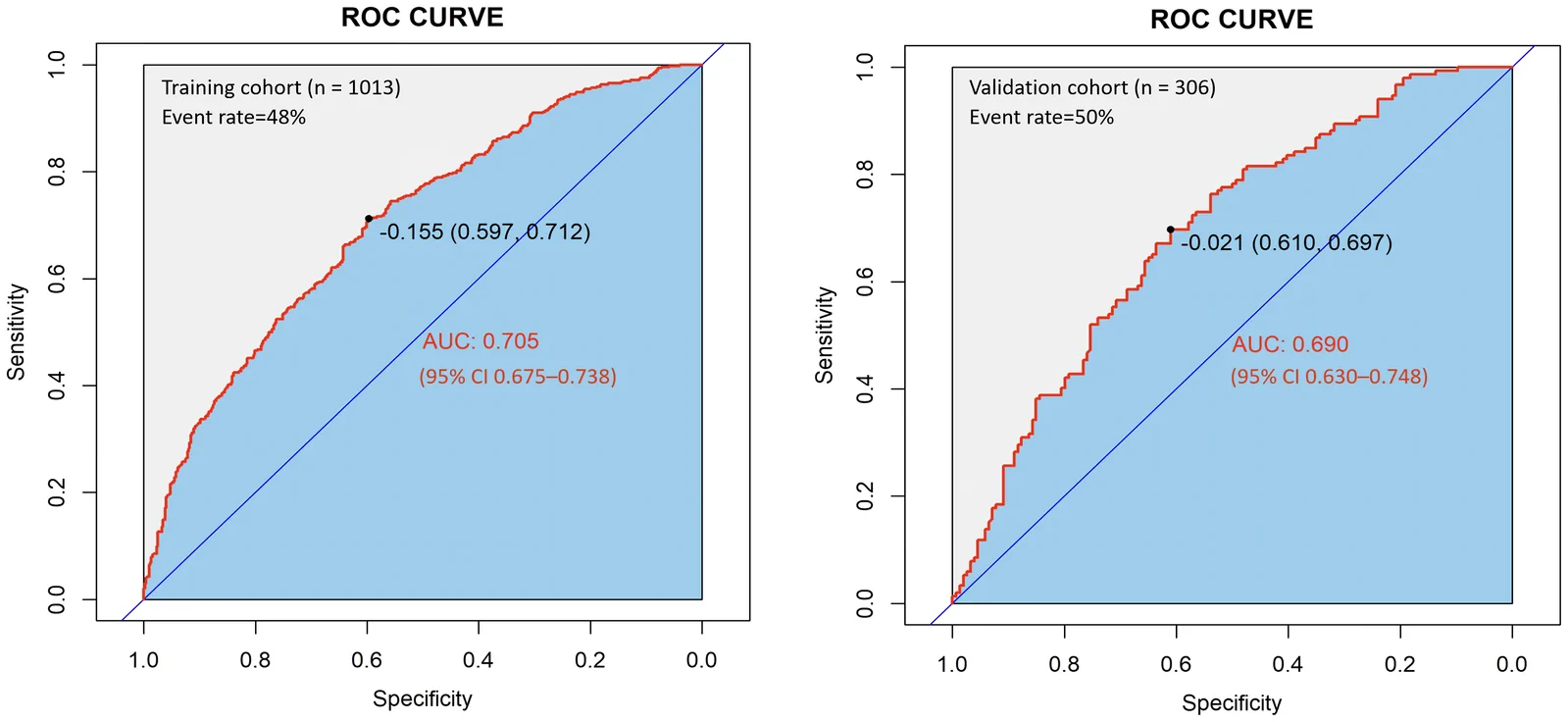
ROC curves for nomogram performance in training and validation cohorts (left: training; right: validation).

### 3.3 Development and interpretation of the XGBoost prediction model

Based on the final variables, an XGBoost prediction model was developed to evaluate clinical pregnancy outcomes following FET, with continuous variables retained in their original form. The final XGBoost model comprised 100 boosting trees with 691 terminal leaves in total, corresponding to a mean of 6.91 terminal leaves per tree, reflecting a depth-constrained boosted-tree structure. The model achieved an AUC of 0.854 (95% CI, 0.832–0.877) in the training cohort and 0.722 (95% CI, 0.666–0.779) in the temporal validation cohort (**Figure 4**), indicating good discriminative ability and acceptable generalizability. Although the XGBoost model showed a numerically higher AUC than the logistic regression model in the temporal validation cohort (0.722 vs. 0.690), the difference was not statistically significant according to DeLong’s test (P = 0.090). The corresponding AUPRC values were 0.840 and 0.707, respectively.

**Figure 4:**
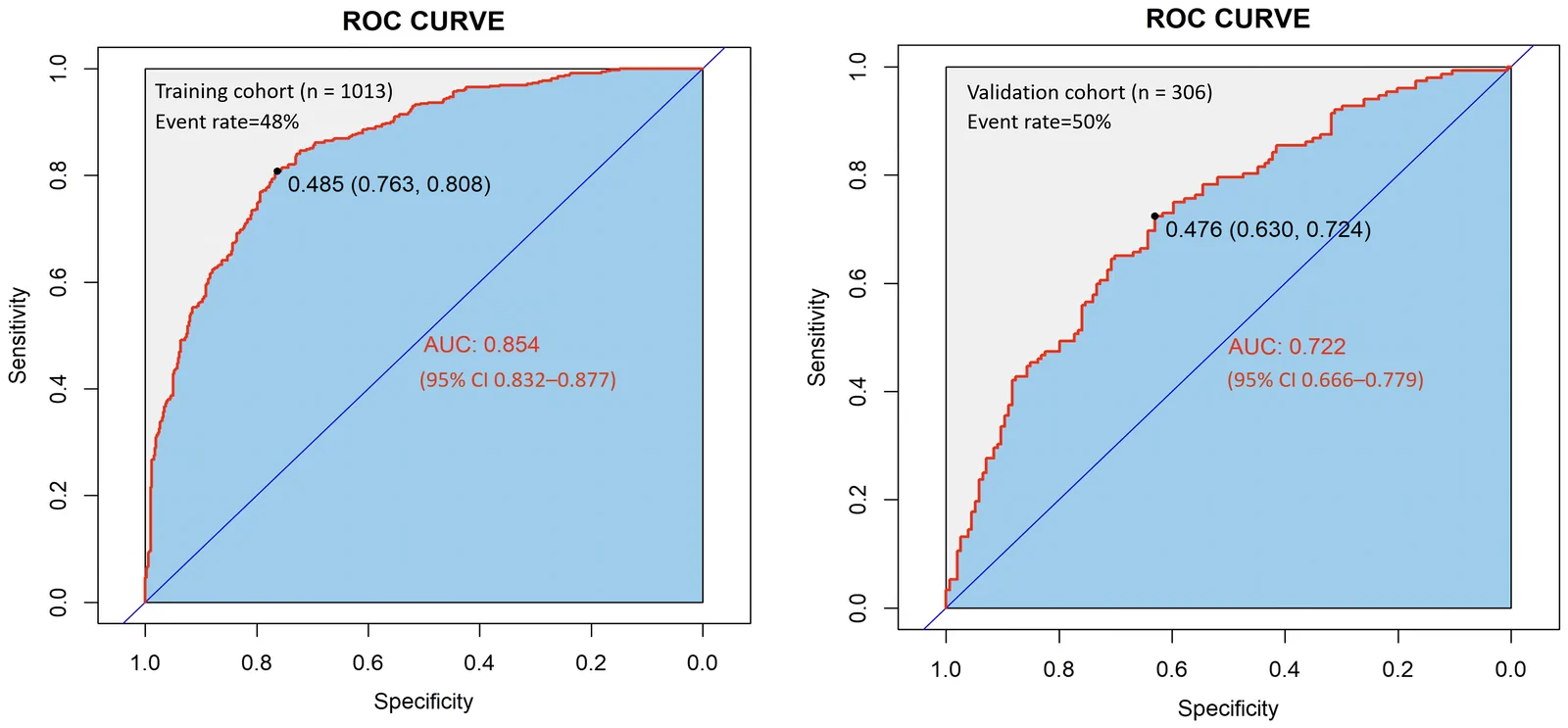
ROC curves for XGBoost model performance in training and validation cohorts (left: training; right: validation).

To interpret the prediction mechanism of the model, the SHAP approach was applied to analyze feature importance and the direction of their effects (**Figure 5A**). The feature importance bar plot indicated that the variables with the greatest impact on pregnancy outcomes were, in descending order, AFC, good-quality embryos, age, endometrial change, cycle type, pre-progesterone ET, retrieval-transfer interval and BMI. Transferred embryos and induced abortions contributed minimally to the model. Partial dependence and Individual Conditional Expectation(ICE) plots for the top continuous predictors (AFC, age, and pre-progesterone ET) are presented in **Supplementary Material (Part 5)** to further visualize their directional effects and possible non-linear patterns.

**Figure 5:**
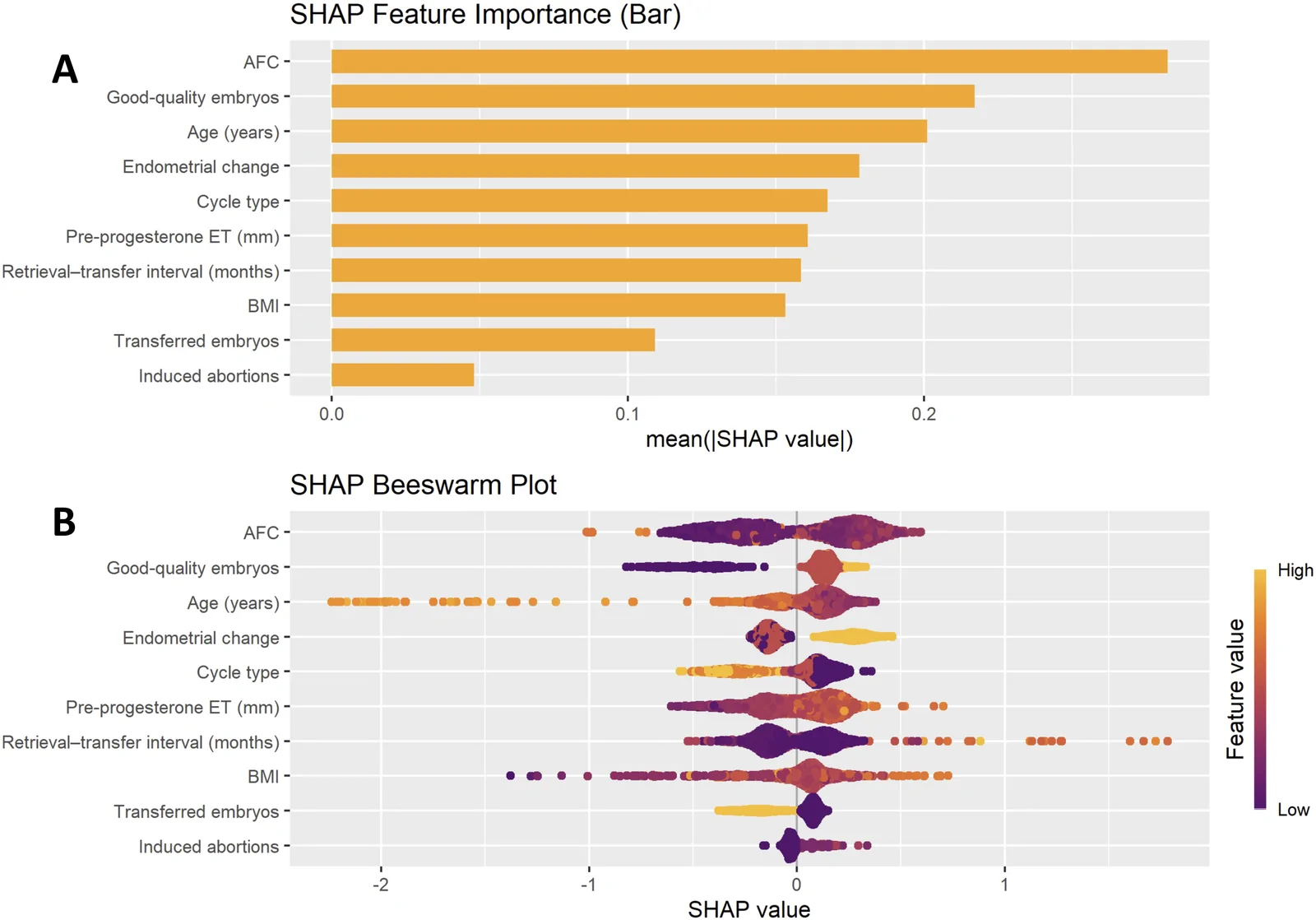
SHAP-based interpretation of the XGBoost model. **(A)** SHAP feature importance bar plot based on the mean absolute SHAP values. **(B)** SHAP beeswarm plot showing the distribution and direction of individual feature effects on model predictions.

The SHAP beeswarm plot (**Figure 5B**) further illustrated the relationships between variable values and predicted pregnancy outcomes. Higher AFC and greater good-quality embryos were associated with larger SHAP values, while age showed a negative association. These directions were broadly consistent with the corresponding logistic regression coefficients, providing a simple concordance check between the two modeling approaches. BMI exhibited a nonlinear pattern, with intermediate values associated with higher predicted probabilities. Endometrial change indicated that stable or moderately increased endometrium was associated with higher predicted pregnancy probability, whereas decrease was associated with lower probability. The retrieval–transfer interval showed a positive association within a certain range but considerable interindividual variability, and the cycle type also exhibited pronounced individual heterogeneity. A higher number of induced abortions tended to be associated with lower predicted probabilities, although the overall impact was limited.

At the individual level, SHAP waterfall plots were further employed to provide case-specific interpretability of the model predictions (**Figure 6**). The same representative patient illustrated using the nomogram was evaluated with the XGBoost model. In this representative case, the model’s raw output value was f(x) = 0.363, corresponding to a predicted pregnancy probability of approximately 59.0% after Sigmoid transformation (13) Details of the transformation method are provided in **Supplementary Material (Part 6)**.

**Figure 6:**
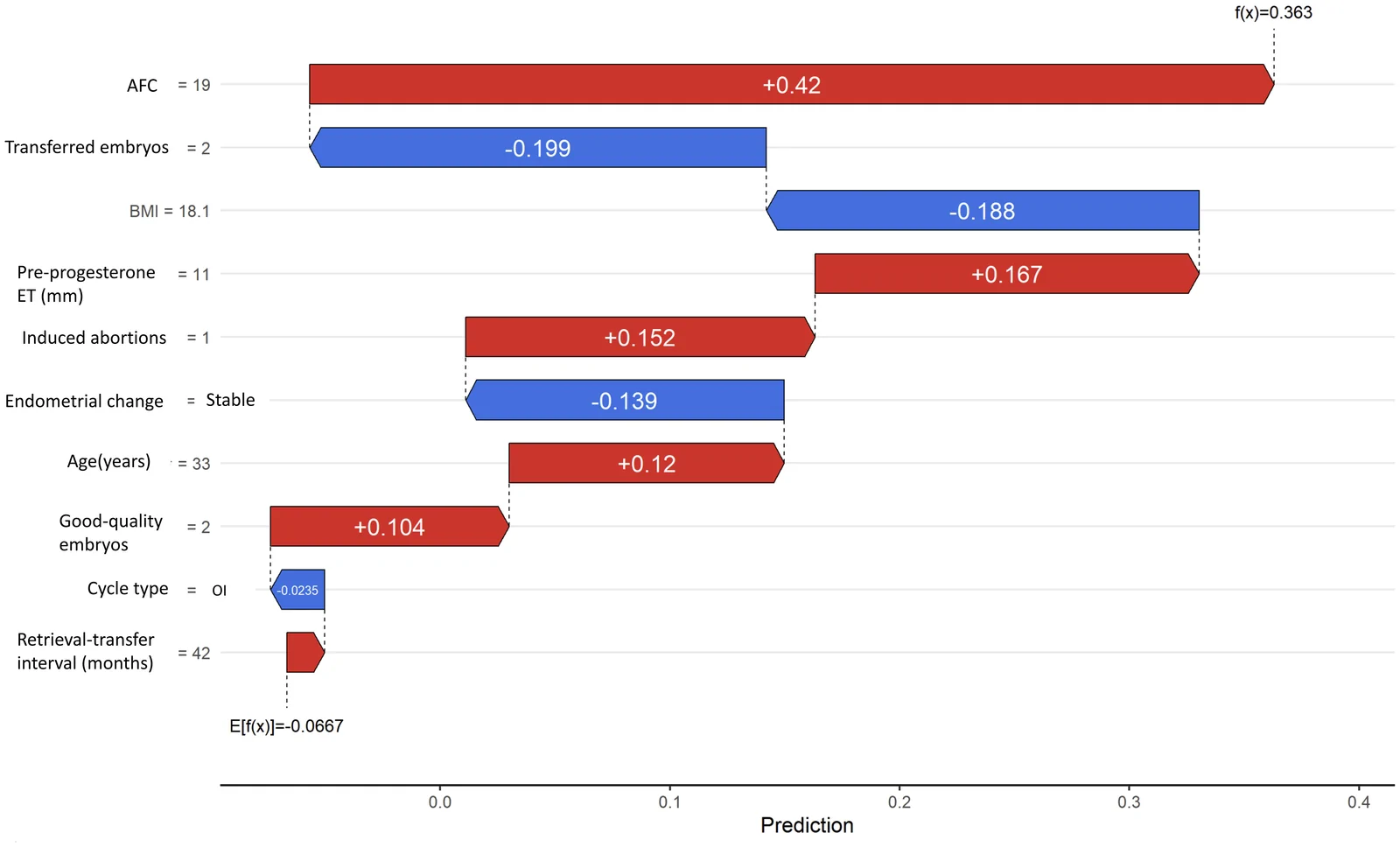
SHAP waterfall plot for individual prediction in the XGBoost model.

### 3.4 Model calibration

Calibration curves for both models are shown in **Figure 7**. In both the training and temporal validation cohorts, the bias-corrected curves were generally close to the ideal diagonal, indicating good agreement between predicted and observed probabilities.

**Figure 7:**
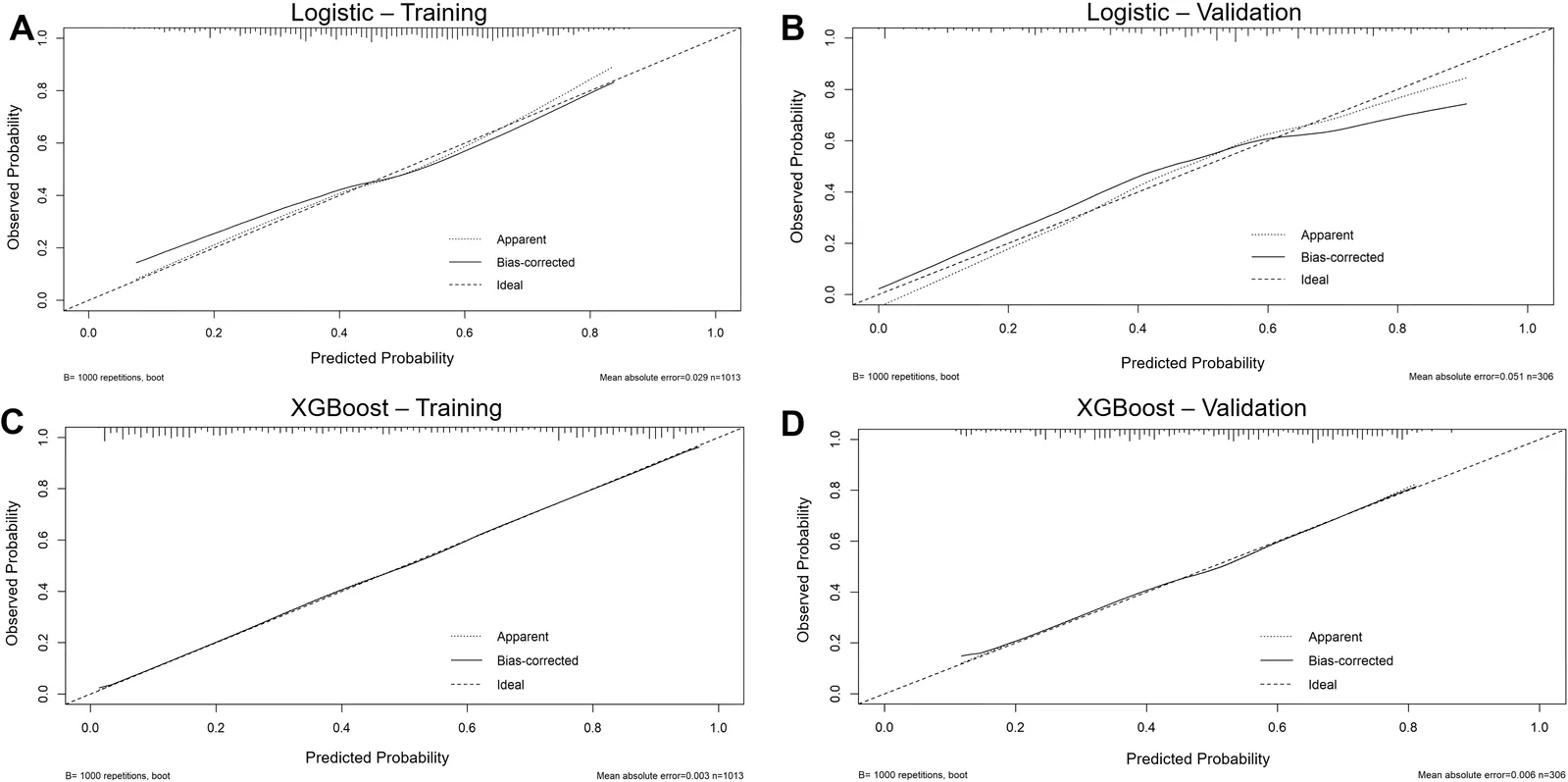
Calibration curves of the logistic regression nomogram and XGBoost models in the training and temporal validation cohorts. [Panels **(A, B)** show the calibration of the logistic regression nomogram in the training and temporal validation cohorts, respectively, whereas panels **(C, D)** show the calibration of the XGBoost model in the training and temporal validation cohorts, respectively].

In the temporal validation cohort, the XGBoost model showed a Brier score of 0.212, calibration intercept of 0.056, and slope of 0.965. The logistic regression model demonstrated similar calibration, with a Brier score of 0.221, intercept of −0.018, and slope of 0.931. These values suggest no substantial miscalibration in either model.

### 3.5 Decision curve analysis for evaluating clinical utility

DCA was performed to further assess the potential clinical utility of the prediction models. In both the training and temporal validation cohorts, the DCA curves of the logistic regression and XGBoost models consistently exceeded the “Treat-All” and “Treat-None” reference lines (**Figure 8**), indicating a positive net benefit across a reasonable range of threshold probabilities. At a clinically representative probability threshold of 0.30, XGBoost showed higher sensitivity than logistic regression in the training cohort (0.969 vs. 0.918), whereas in the temporal validation cohort, logistic regression showed slightly higher sensitivity (0.941 vs. 0.928), indicating a reversal in the relative sensitivity of the two models across cohorts at this threshold. Full threshold-based metrics, including sensitivity, specificity, Positive Predictive Value(PPV), and Negative Predictive Value(NPV), are provided in **Supplementary Material Part 7**. In the temporal validation cohort, compared with a treat-all strategy, the XGBoost model would yield approximately 4 fewer true positives and 15 fewer false positives per 100 patients, while the logistic regression model would yield approximately 3 fewer true positives and 12 fewer false positives per 100 patients, illustrating the clinical trade-off represented in the decision curve.

**Figure 8:**
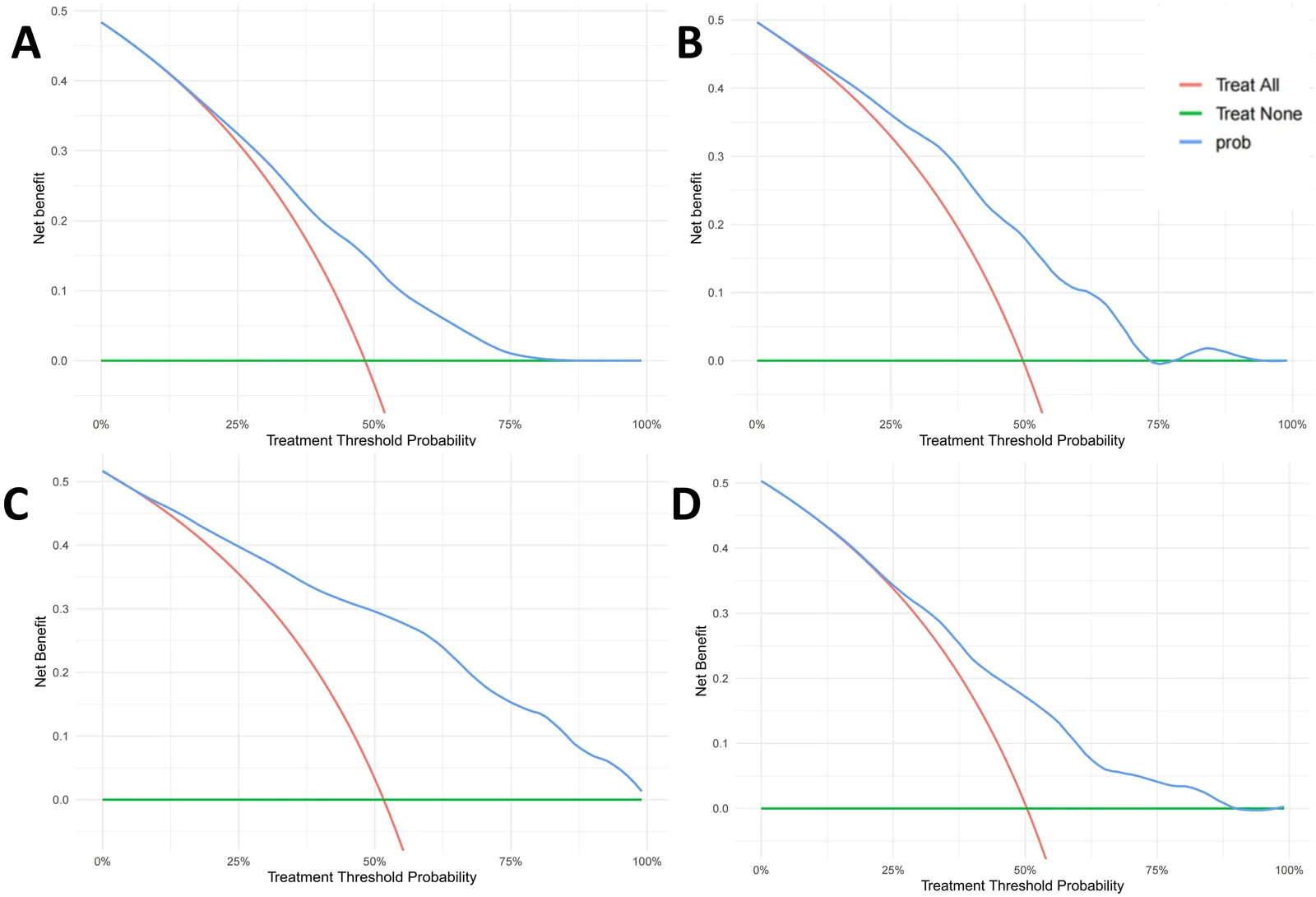
Decision curve analysis of the logistic regression nomogram and XGBoost models. **(A, B)** Logistic regression nomogram (training and validation cohorts); **(C, D)** XGBoost model (training and validation cohorts).

### 3.6 Sensitivity analyses

To ensure a fair comparison with the XGBoost model, two alternative specifications of the logistic regression model were evaluated. First, variables originally categorized based on clinical considerations were entered as continuous linear terms. Second, nonlinear relationships of continuous variables were modelled using restricted cubic splines (RCS). The continuous logistic model showed slightly lower discrimination than the categorized model (C-index: 0.690 vs. 0.705), while the RCS model showed modest improvement (C-index: 0.717), although its discrimination remained numerically lower than that of XGBoost. These findings suggest that performance differences were not primarily driven by variable representation, but may relate to XGBoost’s ability to capture complex nonlinearities and interactions. Considering clinical interpretability and overall performance, the clinically categorized logistic regression model was retained as the primary comparator.

In an additional sensitivity analysis addressing outcome definition, ectopic pregnancies were excluded and both models were re-evaluated. Model discrimination remained essentially unchanged. The logistic regression model achieved AUCs of 0.707 (95% CI, 0.675–0.737) in the training cohort and 0.696 (95% CI, 0.635–0.752) in the temporal validation cohort, while the corresponding AUCs for XGBoost were 0.854 (95% CI, 0.831–0.877) and 0.730 (95% CI, 0.674–0.786), respectively. These findings were consistent with the primary analysis, suggesting that inclusion of the small number of ectopic pregnancies did not materially affect overall model discrimination.

## 4 Discussion

This study developed an explainable XGBoost model based on single-center data to predict clinical pregnancy outcomes following FET cycles. The XGBoost model showed numerically higher discrimination than the conventional logistic regression model, while both models demonstrated acceptable calibration. SHAP analysis identified AFC, age, BMI, ET, and good-quality embryos as key predictive factors. Temporal validation suggested that the model has reasonable temporal transportability and potential clinical applicability. Notably, at the 0.30 threshold, logistic regression showed slightly higher sensitivity than XGBoost in the temporal validation cohort, suggesting that threshold-dependent classification performance may vary across cohorts and should not be interpreted as evidence of consistent superiority of either model.

Compared with previous studies, traditional regression models have shown limited performance in predicting pregnancy outcomes after FET or IVF, with reported discrimination ranging from 0.60 to 0.70 (6–8). Because pregnancy outcomes are influenced by multiple interacting and nonlinear factors, traditional models often fail to capture these complex relationships, thereby limiting predictive accuracy. In contrast, ML approaches can more effectively identify latent patterns and have demonstrated superior performance in reproductive outcome prediction. Several studies have applied and compared multiple ML algorithms-such as random forest, support vector machines, and neural networks- showing substantial improvements in predictive accuracy (9, 20, 21). However, existing models still face notable limitations: many incorporated only a narrow set of clinical variables, failing to fully reflect the multidimensional determinants of pregnancy outcomes; some lacked external validation, leading to suboptimal generalizability; and others suffered from poor interpretability, restricting clinical translation.

In the present study, XGBoost was used as a representative machine-learning approach and combined with SHAP analysis to enhance model interpretability. Beyond the core predictors commonly used in reproductive medicine—such as age, BMI, ET, and good-quality embryos—our model also integrated additional clinical, laboratory, and treatment-related indicators, providing a more realistic representation of the complex process of embryo implantation. On this basis, we employed the XGBoost combined with SHAP analysis to enhance model transparency, achieving both high predictive performance and clinical interpretability. In practical implementation, the model is intended to be applied on the day of frozen embryo transfer, when all required predictors—including transfer-day endometrial thickness—are routinely available, allowing integration into clinical decision-making without reliance on post-transfer information. The intended users of the model are reproductive medicine clinicians, and its application does not require advanced technical expertise beyond routine clinical assessment and data entry. If any predictor is unavailable or of poor quality at the time of use, clinical judgment should prevail, and model predictions should be interpreted cautiously or deferred until reliable measurements are obtained.

The SHAP analysis further revealed each variable’s contribution and direction of effect in the model predictions. Because some predictors may be correlated, SHAP importance rankings should be interpreted cautiously and reflect associations rather than causal effects. As shown by the feature importance and beeswarm plots, AFC emerged as the most influential predictor of clinical pregnancy, with higher AFC levels associated with higher predicted probabilities. However, AFC may partly reflect age or overall reproductive potential rather than exerting a direct causal effect on implantation, particularly in the FET setting where embryo characteristics are already established. In this context, reliance on AFC should be interpreted cautiously, as its influence in the model may be indirect. Accordingly, the model is intended to support probabilistic risk communication rather than to exclude or disadvantage patients with low ovarian reserve who nonetheless have transferable embryos.

In addition, instead of the embryo transfer–day ET commonly used in previous studies, endometrial change and pre-progesterone ET were included in the final model. This combination reflects the dynamic trajectory of the endometrium from preparation to the pre-transfer stage. Physiologically, embryo implantation depends on the temporal coordination between estrogen-induced proliferation and progesterone-mediated receptivity (21). Following progesterone exposure, the endometrium undergoes remodeling accompanied by reduced uterine peristalsis (22, 23). Such dynamic alterations may more accurately reflect synchronization with the implantation window than static endometrial thickness alone. Consistent with the findings, a clinical study by Fangfang Xu et al. (24) reported that endometrial status and its dynamic changes near the implantation window were superior to static thickness measures, while Junqiang Wang et al. (25) also demonstrated that both pre-progesterone ET and embryo transfer–day ET were important predictors of clinical pregnancy. Our results further support the predictive value of dynamic endometrial changes. Given the inherent variability of routine ultrasound measurements, small thickness differences should be interpreted cautiously. Thickness reduction after progesterone exposure may reflect endometrial compaction during secretory transformation, whereas apparent increases may relate to stromal edema, vascular changes, or measurement variability. Accordingly, this variable was used as an objective record of endometrial thickness changes for prediction, rather than as evidence of biological proliferation or atrophy.

Furthermore, a moderate extension of the retrieval–transfer interval appeared to exert a favorable effect on pregnancy outcomes, though considerable individual variability was observed. This phenomenon may be related to avoiding post-stimulation hormonal fluctuations and allowing additional time for endometrial recovery. Previous studies have also reported that embryo cryopreservation within a reasonable time frame does not impair developmental potential (26). However, this does not imply that longer intervals are always better, as time-related factors such as age may counteract the benefit; thus, these findings require further validation in multicenter cohorts. Apart from the above key variables, BMI, good-quality embryos, age, cycle type, and induced abortions also contributed to model predictions, with effect directions generally consistent with clinical experience and previous reports (25, 27). However, the number of patients with two or more prior induced abortions was limited, which may have reduced the model’s ability to fully capture the prognostic contribution of repeated induced abortions and may partly explain the relatively low SHAP importance of this variable. Accordingly, model performance in this higher-risk subgroup could not be reliably evaluated and should be further assessed in larger cohorts with adequate representation of patients with repeated induced abortions.

The XGBoost model maintained moderate discrimination in the temporal validation cohort, although the AUC decreased from 0.854 in the training cohort to 0.722 in temporal validation. Several baseline characteristics differed between the two cohorts, indicating temporal case-mix drift. This distributional shift may have contributed, at least in part, to the observed decrease in discrimination and highlights the importance of evaluating model transportability across different clinical populations and time periods. Given the relatively small size of the temporal validation cohort, the extent to which case-mix differences contributed to the observed decline in discrimination could not be reliably quantified. These findings underscore the need for cautious interpretation of the temporal validation results and for further external validation in independent populations.

This study has several limitations. First, as a single-center retrospective study, it is subject to inevitable selection and information biases, which may limit generalizability. Second, although the model incorporated multiple routinely available clinical variables, it did not include lifestyle factors (e.g., smoking, alcohol consumption) or genetic and molecular biomarkers, potentially omitting additional predictors. Moreover, the model was temporally validated only within our center and lacked external validation across regions and populations, requiring further confirmation of generalizability. Baseline differences between the training and validation cohorts indicate potential case-mix heterogeneity that may partly explain the lower validation AUC. When applied across centers, performance drift may occur due to differences in populations and clinical practice, and local recalibration may be required. Future studies should additionally evaluate performance across clinically and demographically relevant subgroups to confirm robustness and equitable performance. Although SHAP improved interpretability, its values reflect model associations rather than causal effects, and the algorithm’s complexity may limit clinical implementation. The validation results also suggest potential overfitting. In addition, XGBoost was selected as the representative machine-learning approach in the present study. Future multicenter studies with larger and more comprehensive datasets should compare different machine-learning algorithms using standardized development and validation procedures to further improve model robustness and clarify their relative predictive performance.

## 5 Conclusions

This study developed and internally, temporally validated an explainable prediction model integrating XGBoost and SHAP using single-center data to evaluate clinical pregnancy outcomes following FET cycles. The model demonstrated robust discrimination and calibration across both training and internal temporal validation cohorts, offering individualized interpretability to support personalized pregnancy assessment and clinical decision-making. However, as a single-center retrospective study, external validation across multiple regions remains lacking. Future research should validate and refine the model using multicenter, large-scale datasets and develop user-friendly clinical tools to enhance translational application.

## Data Availability

The raw data supporting the conclusions of this article will be made available by the authors, without undue reservation.
