# Supplementary material for: Development and internal temporal validation of an explainable XGBoost model for predicting clinical pregnancy after frozen embryo transfer
Source: Front Endocrinol (Lausanne). 2026 Sep 28;17:1942415. doi: 10.3389/fendo.2026.1942415 (PMC13654733; doi:10.3389/fendo.2026.1942415)
Supplement: Supplementary file 1 [file Supplementaryfile1.docx]

**Part 1 Endometrial Preparation Protocols**

| **Protocol** | **Main Indications** | **Key Procedures / Medications** | **Timing of Endometrial Transformation** | **Timing of Embryo Transfer** |
| --- | --- | --- | --- | --- |
| Natural Cycle (NC) | Regular menstruation with normal ovulatory function | Ultrasound monitoring of follicles and endometrium; urine LH or serum LH/E2 testing; spontaneous ovulation (triggered with hCG if necessary); routine luteal phase support | Confirmation of ovulation | 3–5 days after ovulation(blastocyst) |
| Hormone Replacement Therapy Cycle (HRT) | Irregular menstruation or anovulation; flexible scheduling required | Initiate estrogen (oral or transdermal) on days 3–5 of menses or withdrawal bleeding; once criteria are met, add progesterone for endometrial transformation;routine luteal phase support | Endometrial thickness ≥ 7 mm and E2 > 550 pmol/L (≈ 150 pg/mL) | 3–5 days after progesterone initiation (depending on embryo stage) |
| Ovulation Induction Cycle (OI) | Ovulatory dysfunction | Letrozole or gonadotropins starting from day 3–5 of the cycle; hCG trigger when criteria met;routine luteal phase support | Confirmation of ovulation | 3–5 days after ovulation or hCG trigger |
| GnRH-a-HRT | Adenomyosis or patients requiring pituitary down-regulation | GnRH-a (e.g., goserelin 3.75 mg) administered on day 1–3 of menses; after down-regulation, estrogen is initiated, followed by progesterone once target levels are reached; routine luteal phase support | Endometrial thickness ≥ 7 mm and E2 > 550 pmol/L (≈ 150 pg/mL) | 3–5 days after progesterone initiation |
| GnRH-a-OI | Patients requiring down-regulation and contraindicated for estrogen therapy | Ovulation induction with letrozole or gonadotropins following GnRH-a down-regulation; hCG trigger;routine luteal phase support | Confirmation of ovulation | 3–5 days after ovulation or hCG trigger |

**Part 2 Data dictionary for candidate predictors**

| **Variable** | **Definition** | **Timing** | **Type** | **Unit** | **Coding / Categories** |
| --- | --- | --- | --- | --- | --- |
| Age | Age before FET | Baseline before FET | Continuous | years | Used as continuous |
| BMI | Body mass index | Baseline before FET | Categorized | kg/m² | <18.5; 18.5–<25; 25–<30; ≥30 |
| AMH | Serum anti-Müllerian hormone level | Baseline before FET | Continuous | ng/mL | Used as continuous |
| AFC | Antral follicle count assessed by ultrasound | Baseline ovarian assessment | Categorized | count | <10; 10–<20; 20–<30; ≥30 |
| Duration of infertility | Time from infertility diagnosis to FET | Baseline history | Continuous | years | Used as continuous |
| Infertility type | Primary or secondary infertility | Baseline history | Binary | NA | Secondary infertility; Primary infertility |
| Caesarean section history | Previous caesarean delivery | Baseline history | Binary | NA | 0 = No; 1 = Yes |
| Fertilization method | Fertilization technique used | IVF cycle | Binary | NA | IVF; ICSI |
| Induced abortions | Number of previous induced abortions | Baseline history | Ordinal | NA | 0, 1, 2, 3, ≥4 |
| Cycle type | Endometrial preparation protocol | FET cycle | Categorical | NA | NC; HRT; OI; GnRH-a-HRT; GnRH-a-OI |
| Retrieval-transfer interval | Time between oocyte retrieval and FET | Cycle characteristic | Categorized | months | <10; 10–<30; 30–<60; ≥60 |
| Transferred embryos | Number of embryos transferred | Transfer day | Binary | count | 1; 2 |
| Good-quality embryos | Number of transferred embryos classified as good-quality | Transfer day | Ordinal | count | 0; 1; 2 |
| Pre-progesterone ET | Endometrial thickness before progesterone initiation | Pre-progesterone day | Categorized | mm | <7; 7–<10; 10–<12; ≥12 |
| Transfer-day ET | Endometrial thickness on embryo transfer day | Transfer day | Categorized | mm | <8; 8–<11; 11–<15; ≥15 |
| Endometrial change | Difference between transfer-day ET and pre-progesterone ET | Derived variable | Categorical | NA | Decrease; Stable; Increase |
| PCOS | Diagnosis of polycystic ovary syndrome | Baseline diagnosis | Binary | NA | 0 = No; 1 = Yes |
| Endometriosis | Clinical diagnosis of endometriosis | Baseline diagnosis | Binary | NA | 0 = No; 1 = Yes |
| PID sequelae | Sequelae of pelvic inflammatory disease | Baseline diagnosis | Binary | NA | 0 = No; 1 = Yes |
| Adverse reproductive history | History of miscarriage or reproductive complications | Baseline history | Binary | NA | 0 = No; 1 = Yes |
| DOR | Diminished ovarian reserve diagnosis | Baseline assessment | Binary | NA | 0 = No; 1 = Yes |
| Tubal abnormality | Tubal factor infertility or abnormality | Baseline diagnosis | Binary | NA | 0 = No; 1 = Yes |
| Pregnancy status | Clinical pregnancy outcome | Follow-up ultrasound | Binary | NA | 0 = No pregnancy; 1 = Clinical pregnancy |

**Part 3 XGBoost hyperparameters and tuning**

The XGBoost model was trained using the training cohort with a conservative hyperparameter configuration to limit model complexity. The final settings were: learning rate (eta) = 0.1, max_depth = 3, and number of boosting rounds (nrounds) = 100. Other parameters were kept at their default values.

**Part 4 Flowchart of patient selection and cohort derivation.**

**Part 5 Partial dependence and ICE plots for the top continuous predictors in the XGBoost model**

The red curves represent the average partial dependence, indicating the overall marginal relationship between each predictor and the predicted probability of clinical pregnancy. The black lines represent individual conditional expectation (ICE) curves, illustrating interindividual variability in model predictions. Panels show female age (left), antral follicle count (AFC, middle), and pre-progesterone endometrial thickness (right). These plots demonstrate potential nonlinear associations and heterogeneous prediction patterns across patients.

**Part 6 Conversion of Model Output to Predicted Probability**

The original model output value f(x) was transformed into the predicted probability of clinical pregnancy using the Sigmoid function, as follows:

where P represents the predicted probability, and f(x) denotes the raw model output.

This transformation method is commonly applied in both traditional statistical models (such as logistic regression) and machine learning classifiers (such as XGBoost) to convert raw model outputs into probability values, thereby reflecting the likelihood of an event occurring for a given individual.

**References**

Hosmer DW, Lemeshow S. Applied Logistic Regression. 2nd Edition ed. New York: Wiley; 2000.

Lundberg SM, Lee S. A unified approach to interpreting model predictions. In: Editor, editor NIPS'17; 2017; Red Hook, NY, USA. Pub Place; 2017. p. 4768-77.

**Part 7 Threshold-based classification performance at probability threshold = 0.30**

**Training cohort**

| Model | Sensitivity | Specificity | PPV | NPV | Accuracy | Balanced Acc |
| --- | --- | --- | --- | --- | --- | --- |
| XGBoost | 0.969 | 0.356 | 0.585 | 0.925 | 0.653 | 0.663 |
| Logistic | 0.918 | 0.275 | 0.543 | 0.783 | 0.586 | 0.597 |

**Temporal validation cohort**

| Model | Sensitivity | Specificity | PPV | NPV | Accuracy | Balanced Acc |
| --- | --- | --- | --- | --- | --- | --- |
| XGBoost | 0.928 | 0.292 | 0.564 | 0.804 | 0.608 | 0.610 |
| Logistic | 0.941 | 0.240 | 0.550 | 0.804 | 0.588 | 0.591 |
